# Prediction of Thermal Damage upon Ultrafast Laser Ablation of Metals

**DOI:** 10.3390/molecules26216327

**Published:** 2021-10-20

**Authors:** Liliana Cangueiro, José Antonio Ramos-de-Campos, David Bruneel

**Affiliations:** LASEA, Liège Science Park, Rue Louis Plescia 31, 4102 Seraing, Belgium; jaramos@lasea.com (J.A.R.-d.-C.); dbruneel@lasea.com (D.B.)

**Keywords:** ultrafast laser processing, heat accumulation, femtosecond laser

## Abstract

Ultrafast lasers micromachining results depend on both the processing parameters and the material properties. The obtained thermal effects are negligible if a good combination of processing parameters is chosen. However, optimizing the processing parameters leading to the required surface quality on a given material can be quite complex and time consuming. We developed a semi-empirical model to estimate the heat accumulation on a surface as a function of the laser fluence, scanning speed and repetition rate. The simulation results were correlated with experimental ones on different materials, and compared with the transient temperature distributions calculated using an analytical solution to the heat transfer equation. The predictions of the proposed model allow evaluating the heat distribution on the surface, as well as optimizing the ultrafast laser micromachining strategy, yielding negligible thermal damage.

## 1. Introduction

It has been thoroughly demonstrated in the past years that ultrafast lasers are excellent tools for ablating and micromachining virtually all types of materials [1]. They allow cutting, milling, etching and texturing surfaces with micrometer precision control, and the resulting thermal effects are negligible, provided that an optimal set of processing parameters is used. Depending on the material and its thermal properties, if the processing parameters used are not good, typically by combining high fluences with high repetition rates and/or low scanning speeds, often the thermal damage can jeopardize the laser application, due to the accumulation of residual heat in the surface regions remaining after ablation [2]. If there are already existing tools (i.e., LS-Plume^®^ [3]) that can predict resulting ablation profiles, the prediction of thermal damages is a very active research field and of high interest to be implemented in such a tool.

The extent of the thermal effects obtained upon laser irradiation will depend on the processing parameters and the thermal properties of the material. Provided that the material has time to cool down in between pulses, as is the case when working at relatively low repetition rates, the radiation fluence is one of the processing parameters playing a major role in this process, because it determines the ablation mechanism taking place, which, in turn, will determine the morphology, composition and properties of the ablation surface [4,5,6]. When working at high repetition rates, cumulative thermal effects take place because the heat conduction characteristic times, of the order of the microsecond, do not allow the material to cool down in between pulses. In addition, particle or plasma-shielding effects may have to be considered because the ablated material ejection plume and associated shock waves are still active and close to the target up to nano- to microseconds after the laser pulse and may prevent the absorption of the subsequent laser pulses [7,8,9]. This behavior is often assumed to be behind the higher ablation rates obtained when working with odd rather than even numbers of pulses in the (MHz) bursts [10,11,12,13,14,15].

Due to the complexity and non-linear nature of the ultrashort pulsed laser-material interaction, determining the optimal processing parameters leading to acceptable thermal effects and the best cycle times required in industry is often a time- and material-consuming process. There are some approaches found in the literature that allow determining the approximate temperatures reached at the irradiated material surface. Some are based on the combination of molecular dynamics and the transfer of energy from the hot electrons that absorb the laser radiation in the first place, to the cold lattice, on the basis of the two-temperature model [16,17,18,19]. The results obtained are of great help to understand the transient laser-material interaction processes, the mechanisms of ablation, and the extent of thermomechanical damage within the ablation surface. However, those calculations require knowing very well a range of thermophysical properties of the material, which are difficult to determine if we are to study a heterogeneous material, such as an alloy, for instance, not to mention the computational power required to simulate the tens of overlapped pulses often used for femtosecond laser micromachining.

Other heat simulation approaches are simpler and aim at describing what happens after ablation. In fact, the energy absorption by the electrons subsystem and consequent transfer of this energy to the lattice subsystem occurs during the first couple of picoseconds after the laser pulse. Once the electron and the lattice subsystems reach thermal equilibrium, and depending on the local temperature and pressure at stake, ablation may take place via liquid spallation or phase explosion, and this takes place after a few tens to hundreds of picoseconds. However, the conversion of absorbed energy to ablation is often not total, and the hot layer at the surface of the workpiece that was not ejected or vaporized then transfers its thermal energy to the bulk, in the range of nano to microseconds. This residual thermal energy can be quantified using a calorimetric setup, and accounts to about 20 to 70% of the incident energy [20,21,22]. The residual heat conduction through the material can then be analyzed within the framework of classic thermodynamics. This approach is widely used to describe the macroscopic thermal effects resulting after ablation [20,23,24]. In general, authors treat this heat source as an instantaneous, pulsed one following the laser radiation deposition [18,23,24]. Alternatively, Bornschlegel et al. [25] used a finite element simulation tool to estimate the temperature distribution upon high power ultrashort pulsed laser ablation considering that the heat source is actually a continuous one of 30% of the average power. The obtained results match with the experimental ones rather well, especially when compared to the ones obtained at 6.5 MHz. Rahaman et al. [26] developed an analytical solution to the heat transfer equation to described both ablation and thermal effects upon ultrafast laser processing of polypropylene, the simulated results being in good agreement with the experimental ones, although this solution is quite complex mathematically.

The objective of this work consists of evaluating and demonstrating a semi-empirical model, predicting thermal damage on ablation surfaces produced on metals with ultrafast laser treatment. This is carried out by estimating the relative amount of energy deposited on the surface of the workpiece and directly correlating it with experimental observations of the thermal damage on the metals obtained, using a wide window of processing parameters.

## 2. Materials and Methods

The ablation experiments performed were carried out on polished stainless steel AISI 316L, aluminum 2024-T3 alloy and Cu samples. The tests were carried out in air, using a Satsuma HP${}^{2}$ femtosecond laser from Amplitude with pulse duration of about 280 fs, 1030 nm radiation wavelength and maximum power of 20 W at 500 kHz. The pulse energy and beam expansion factor were controlled with an motorized beam expander from Lasea. The beam was focused at the surface of the samples, using a 100 mm focal length telecentric lens, yielding, depending on the expansion factor, spot radii of 7 μm and 13.5 μm, determined using the D${}^{2}$ method proposed by Liu et al. [27]. Samples were ablated using pulse energies in the range between 0.3 μJ and 15 μJ at pulse repetition rates of 100 kHz and 500 kHz. The treated samples were ultrasonicated for about 5 min in isopropanol prior to the surface analysis. The microscopic morphological characterization of the treated samples was carried out by electron microscopy using a JEOL JSM-6610, whereas for the macroscopic analysis, we took photos of the samples using a Nikon D90. The surface roughness of the samples was analyzed, using a confocal laser scanning microscope (Olympus LEXT OLS4100).

## 3. Heat Accumulation Models

Two approaches were used to estimate the extent of the thermal damage upon laser treatment of the metals: the first one based on the heat accumulation model proposed by Bauer et al. [23], and the second one on the basis of a semi-empiric analysis of the total energy deposition.

### 3.1. Heat Accumulation Model

The first approach considers that part of the absorbed pulse energy ($E_{P}$) is used for ablation if the pulse energy exceeds the ablation threshold, but in the regions where the absorbed energy is insufficient for ablation to take place, other heat-induced phase transformations may occur. The depth of material that is thermally affected depends on the deposited thermal energy $E_{thermal}$ and on the thermal properties of the material.

The temperatures reached at the workpiece after irradiation with an ultrashort laser pulse with energies above the ablation threshold can be estimated if we take into account that the heat is concentrated in the most superficial layer of the ablation surface in a time scale shorter than the time required for heat conduction to the bulk. This residual thermal energy can then be considered an instantaneous superficial point heat source with energy *Q*, and the temperature distribution below this surface after a single laser pulse can be calculated by applying the heat conduction equation. Considering that the thermal properties of the solid are temperature independent, the temperature distribution within a semi-infinite homogeneous solid is given by the following:

(1) $$T\left(r,t\right)=\frac{Q}{\rho c_{p}{\left(4\pi \kappa t\right)}^{3/2}}e^{-\frac{r^{2}}{4\pi t}}$$

where *r* is the distance from the point source, *t* the time after the energy pulse, $c_{p}$ the material average heat capacity, ρ the mass density, and κ the thermal diffusivity [28]. The solution for a Gaussian surface heat source can be obtained by integration of this equation, resulting in the following equation for the temperature distribution:

(2) $$\begin{array}{c} T\left(x,y,z,t\right)=\frac{2E_{thermal}}{\pi \rho c_{p}\sqrt{\pi \kappa t}\left(8\kappa t+\omega_{0}^{2}\right)}\\ \;\;\;\;\;\;\;\;\;\times \exp\left[\frac{{\left(x-x_{c}\right)}^{2}+{\left(y-y_{c}\right)}^{2}}{4\kappa t}\left(\frac{\omega_{0}^{2}}{8\kappa t+\omega_{0}^{2}}-1\right)\right]\exp\left[\frac{-z^{2}}{4\kappa t}\right] \end{array}$$

In this equation, $\left(x-x_{c}\right)$, $\left(y-y_{c}\right)$ and *z* represent the distance from the center of the laser spot $\left(x_{c},y_{c},0\right)$, and $\omega_{0}$ is the beam radius at 1/e of the maximum intensity. The temperature at the center of the laser spot $\left(x=x_{c},y=y_{c}\right)$ as a function of the depth and time is given by the following:

(3) $$T\left(x_{c},y_{c},z,t\right)=T_{offset}+\frac{2E_{thermal}}{\pi \rho c_{p}\sqrt{\pi \kappa t}\left(8\kappa t+\omega_{0}^{2}\right)}e^{-\frac{z^{2}}{4\kappa t}}.$$

For multi-pulse irradiation, the time distance between pulses is $t_{\left(p-p\right)}=1/PRR$, where PRR is the pulse repetition rate. The temperature distribution after *N* pulses is thus given by the following:

(4) $$T\left(x,y,z,t\right)=\sum_{n=0}^{N}T_{x_{c_{n}},y_{c_{n}}}^{SinglePulse}\left(x,y,z,t+nt_{p-p}\right)$$

In order to perform these calculations, the percentage of the pulse energy that remains in the material after ablation used was 28% [23]. The material thermal properties used in the calculation, listed in Table 1, were assumed to be independent of the temperature. We considered that the material was at an offset (room) temperature ($T_{offset}$) of 20 °C.

**Figure 1:**
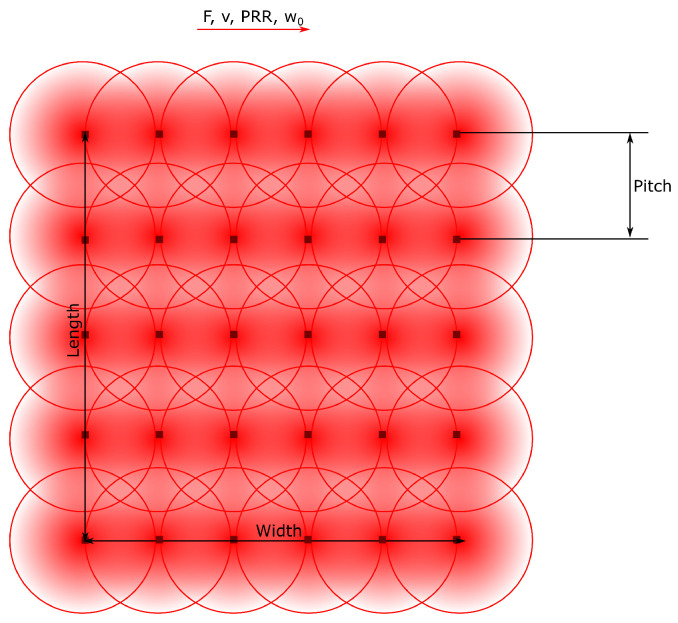
Disposition of points at which the temperature is determined over an area produced by scanning the beam over parallel lines distanced by a pitch, with a certain fluence, scanning speed and pulse repetition rate.

This approach was used to determine the maximum temperature reached per point on a treated surface produced by hatching parallel laser lines distanced by a certain pitch with a determined speed and repetition rate, as illustrated in Figure 1. We considered that heat propagates from the surface to the infinitely deep bulk of the solid, equally in all directions (x,y,z). To these lines, we added the time delays corresponding to the acceleration and deceleration ramp times at the end and beginning of each line, respectively. This is the typical behavior of a beam moved in relation to the specimen when a galvo scanner or mechanical stages are used.

### 3.2. Semi-Empirical Heat Absorption Estimation

The second approach used to estimate the extent of thermal degradation upon ultrafast laser ablation is a much simpler one and considers that the accumulation of heat is closely related to the rate of energy deposition on the surface or irradiance. According to Rahaman et al. [26], the response of the material after absorbing ultrashort laser pulses depends on the absorbed laser intensity $I_{a}$, which can be described as follows:

(5) $$I_{a}=\frac{2AE_{P}PRR}{\omega_{0}v\tau_{P}},$$

where *A* is the absorptivity of the workpiece, *v* the scanning speed and $\tau_{P}$ the pulse width. Furthermore, thermal damage is caused by the excessive accumulation of heat, which is not enough to evaporate the material, cannot efficiently diffuse out of the ablation region in between consecutive laser pulses and after irradiation, and is sufficient to induce phase transformations and cool down molten material. The total thermal energy absorbed per surface point upon irradiation with a moving Gaussian beam can be written as follows:

(6) $$Q=\sum_{n=-N}^{N}\frac{4E_{thermal}}{\pi \omega_{o}^{2}}\exp\left(-2\frac{{\left(xn\Delta x\right)}^{2}+y^{2}}{\omega_{o}^{2}}\right),$$

where *N* is the number of overlapped pulses per spot at the center of a line track:

(7) $$N=\frac{2w_{o}\times PRR}{v}.$$

**Figure 2:**
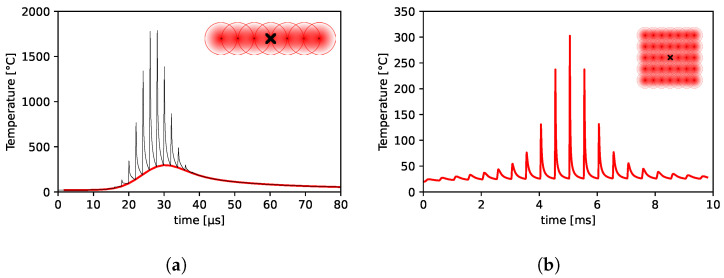
(**a**) Temperature distribution at the center of a 100 μm long lased line (see schematic illustration at the top right corner) on stainless steel, using 2 J/cm^2^ peak fluence, 1 m/s scanning speed at 500 kHz repetition rate. The black plot has all the time points plotted, showing the temperature evolution resulting from each laser pulse, while the red line plots just the corresponding local minima. (**b**) Temperature distribution (at the ablation surface) at the center of an ablated square of 100 μm × 100 μm (see schematic illustration at the top right corner), using the same laser parameters as in the line simulation on the left, with 5 μm pitch and considering 400 μs acceleration plus deceleration delays.

When metals are irradiated with ultrashort laser pulses, heat accumulation leading to thermal damage takes place once the ablated material is ejected away. We consider this a background, continuous process of heat absorption, as the characteristic heat diffusion distance ($2\sqrt{\kappa t}$) for a metal is of the same order of magnitude of the pulse-to-pulse distance Δx. This means that a point at the center of an irradiated line may not have the time to efficiently cool down in between pulses, as illustrated in the temperature distribution plot in Figure 2. If the rate of absorbed heat exceeds the rate at which this heat can diffuse out of the ablation region, the temperature gradient generated established within the bulk can allow phase transformations to take place.

The total absorbed energy converted into heat $I_{thermal}$ can be correlated to the total amount of absorbed energy that was not directly converted into ablation, the scanning speed and the pulse-to-pulse distance, as follows:

(8) $$I_{thermal}\propto \frac{Q}{v\Delta x}.$$

We denote this parameter thermal damage criterion, $C_{td}=\frac{Q}{v\Delta x}$, in J·s, as an equivalent to the summation of heat over time. This way, not only do we look at how much energy is absorbed by the surface, but we also give extra relevance to the amount of time during which the surface is exposed to heat.

## 4. Results and Discussion

The plot in Figure 2a shows the temperature distribution, using the first approach described above in Section 3.1, at the center of a 100 μm long lased line on stainless steel, using 2 J/cm^2^ peak fluence, 1 m/s scanning speed at 500 kHz repetition rate and 28% residual heat. The plot shows that the material cannot cool down in between pulses, resulting in an accumulation of heat in the ablation surface. If the accumulated temperature before the subsequent pulse, corresponding to the local minima in the plot represented by the red dashed line, exceeds a critical or modification temperature of the material, thermal damage will take place [23,29]. The maximum of these local minima is thus often interpreted as the maximum temperature reached by the ablation surface. The plot in Figure 2b shows the temperature distribution at the center of an ablated square of 100 μm × 100 μm, using the same laser parameters as in the point described by Figure 2a, with 5 μm pitch and considering 400 μ s acceleration plus deceleration delays at the end of each scanned line. In this figure, only the local minima, or the time points at which each pulse heats up the surface (analogous to the red dashed line in Figure 2a), are plotted for an easier lecture of the data. These results illustrate the influence of the processing delays and neighboring ablated lines in the accumulated heat at each surface point. Different maximum temperatures are expected should the area dimensions vary.

In order to evaluate the influence of different processing parameters, namely the peak fluence, scanning speed, pitch or spot size, and to determine the modification temperature of the material and the optimal parameters’ window for the laser treatment, we simulated both the maximum temperatures reached and accumulated heat, using several combinations of parameters, and compared the results with the experimental ones. Depending on the application, often the quality requirement is a visual one and/or topographical one, and the objectives are to obtain surfaces that are not macroscopically (or visually) degraded and present low roughness. For instance, obtaining low roughness is of utmost importance in three-dimensional micromachining applications. Therefore, we compare the simulated data with photographs of the corresponding treated areas, and show an example of the evolution of the surface roughness for one of situations tested on stainless steel.

**Figure 3:**
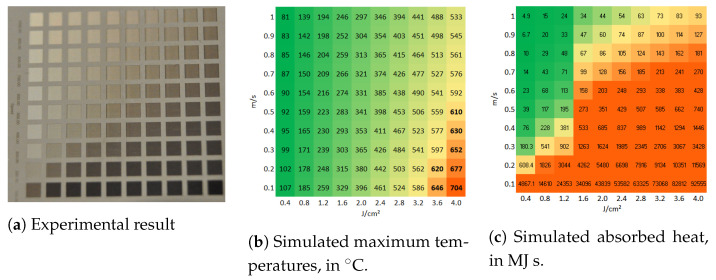
(**a**) Matrix of ablated areas produced stainless steel 316L using peak fluences in the range between 0.4 J/cm^2^ to 4 J/cm^2^ (left to right) and scanning speeds varying from 0.1 m/s to 1 m/s (bottom to top) at 500 kHz pulse repetition rate, 14 μm spot diameter at focus, 5 μm pitch and 5 layers. The area of each ablated square is 1.5 mm × 1.5 mm. (**b**) The simulated temperatures’ colors were chosen so that areas at which the temperature exceeds 600 °C are orange. (**c**) The simulated absorbed heat colors chosen are yellow if $C_{td}$ > 70 MJ s and orange if $C_{td}$ > 200 MJ s (and green otherwise) for fluences above 1.6 J/cm^2^ (high fluence range), and yellow if $C_{td}$ > 400 MJ s and orange if $C_{td}$ > 2000 MJ s for lower fluences, or the low fluence range.

**Figure 4:**
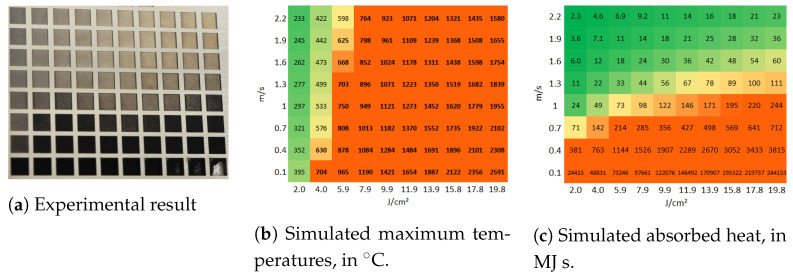
(**a**) Matrix of ablated areas produced on stainless steel 316L using peak fluences in the range between 2 J/cm^2^ to 19.8 J/cm^2^ (left to right) and scanning speeds varying from 0.1 m/s to 2.2 m/s (bottom to top) at 500 kHz pulse repetition rate, 14 μm spot diameter at focus, 5 μm pitch and 50 layers. The area of each ablated square is 1.5 mm × 1.5 mm. (**b**) The simulated temperature color is orange if the temperature exceeds 600 °C. (**c**) The simulated absorbed heat color is yellow if $C_{td}$ > 70 MJ s and orange if $C_{td}$ > 200 MJ s, and green otherwise.

Figure 3a presents a matrix of ablated areas produced on stainless steel 316L, using peak fluences in the range between 0.4 J/cm${}^{2}$ and 4 J/cm${}^{2}$ and scanning speeds varying from 0.1 m/s to 1 m/s at 500 kHz pulse repetition rate, 14 μm spot diameter at focus, 5 μm pitch and 5 layers. Figure 4a presents a matrix of ablated areas produced on the same metal, using peak fluences in the range between 2 J/cm${}^{2}$ and 19.8 J/cm${}^{2}$ and scanning speeds varying from 0.1 m/s to 2.2 m/s at the same repetition rate as the matrix depicted in Figure 3a, as well as the same spot diameter, pitch and 50 of layers. Figure 3b and Figure 4b show the corresponding simulated maximum temperatures reached, considering 28% residual heat, with the colors green-yellow-orange associated with low to high temperatures to facilitate the analysis of the table. In turn, Figure 3c and Figure 4c list the corresponding estimated absorbed heat values $C_{td}$, in green-yellow-orange colors, in which the areas are yellow for $C_{td}>70\;MJ\;s$, orange if $C_{td}>200\;MJ\;s$ and green for lower values, for fluences higher than 1.5 J/cm^2^. For lower fluences, the areas are yellow for $C_{td}>400\;MJ\;s$, orange if $C_{td}>2000\;MJ\;s$ and green otherwise.

**Figure 5:**
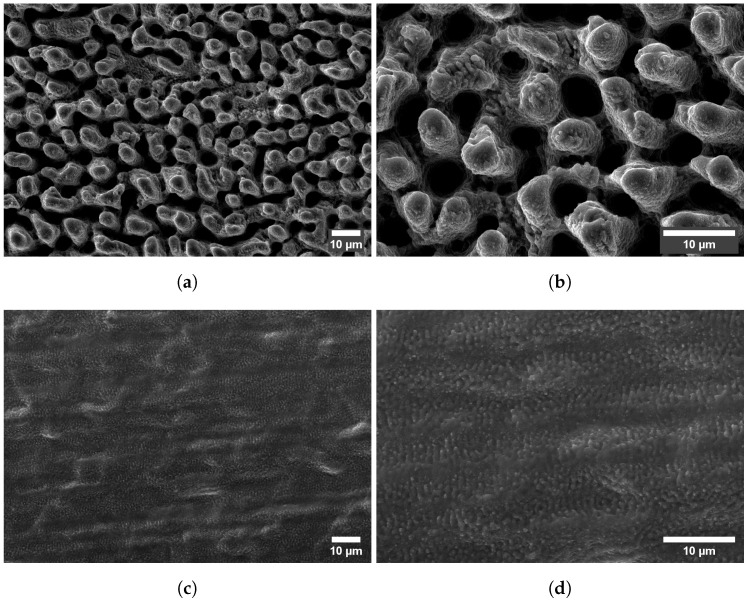
SEM micrographs of the areas ablated on stainless steel 316L using the fluences and scanning speed indicated in each micrograph, at 500 kHz pulse repetition rate, 14 μm spot diameter at focus, 5 μm pitch and 5 layers. (**a**) 4 J/cm^2^, 0.7m/s, 1000× magnification. (**b**) 4 J/cm^2^, 0.7m/s, 2500× magnification. (**c**) 15.8 J/cm^2^, 2.2m/s, 1000× magnification. (**d**) 15.8 J/cm^2^, 2.2m/s, 2500× magnification.

**Figure 6:**
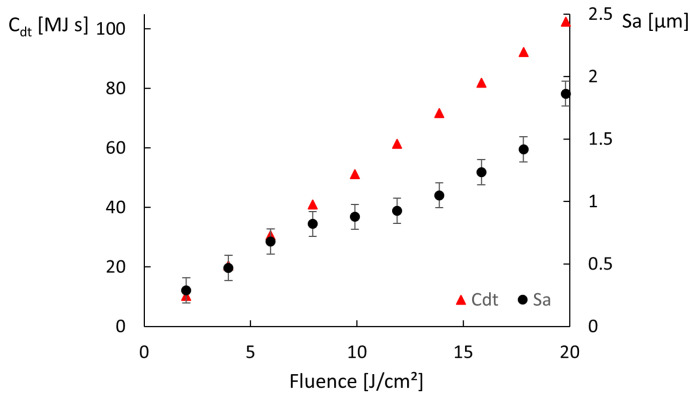
$C_{td}$ and surface roughness Sa evolution with increasing fluence for areas produced on stainless steel with 1.3 m/s scanning speed, 14 μm spot diameter at 500 kHz.

These results show that, for these ranges of speeds and fluences, combining high fluence with low scanning speed results in the accumulation of heat by the ablation surface, leading to visible thermal degradation. Regarding the corresponding simulated temperatures, they increase with increasing fluence and decreasing scanning speed as expected, but a single of threshold modification temperature above which thermal damage is observed cannot be determined. If we compare solely the results of a single line (constant speed, increasing fluence) or column (constant fluence, increasing speed) of one of the matrices, we verify that above a certain fluence or below a certain speed, the ablation surface is thermally affected. However, the simulated temperature at which such modification takes place is not systematic or independent of the parameters. For example, in the first matrix (Figure 3), the treated area is damaged for 0.8 J/cm^2^ and 0.1 m/s and undamaged when using 1.2 J/cm^2^ and 1 m/s, but the estimated temperatures are 194 °C and 185 °C, respectively, so quite comparable, contrarily, to the obtained morphologies. Additionally, the treatments performed at high fluences and high scanning speeds yield surfaces free of thermal damage, as can be seen in the matrix depicted in Figure 4a or the micrographs in Figure 5c,d, for which the simulated maximum temperature was 1321 °C. The maximum temperature obtained for the conditions at which the area depicted in Figure 5a,b was 576 °C, which is lower than the heat modification temperature threshold indicated by Bauer et al. [23], though the macro- and micrographs show a rough and porous surface with a coral structure, described in the literature as an effect of heat accumulation [5,30]. The graph in Figure 6 plots the values of the surface roughness Sa of the treated specimens as well as the corresponding values of $C_{td}$ for areas produced on stainless steel with 1.3 m/s scanning speed, 14 μm spot diameter at 500 kHz (corresponding to the forth line of areas depicted in the matrix in Figure 4). The lower slope of Sa above 10 J/cm^2^ may indicate that the surface was partially molten upon the laser treatment [31]. Still, these results show the proportionality of the two parameters, $C_{td}$ and Sa, and validate the first one. Regarding the heat values calculated, using the approach described in Section 3.2, the results demonstrate that, for this range of processing parameters, the results correlate with the experimental ones for both matrices, at fluences above ≈$1.5\;J/{\mathrm{cm}}^{2}$, and the value of 200 MJ s can be used as criteria to predict thermal damage, even for fluences above 10 J/cm^2^. For fluences lower than about 1.5 J/cm^2^, this heat threshold of 200 MJ s above which we expect thermal damage is underestimated. If this threshold is rather 2000 MJ s, the correlation between the calculation and the experimental results improves. This can be explained by the existence of two different ablation regimes. For the regime prevailing at low fluences, the ablation depth is determined by the optical penetration depth of the material. On the other hand, at high fluences, the energy transport is dominated by the electronic heat diffusion during the laser pulse, which is too low for low fluences, but increases at higher electron temperatures reached at high laser fluences [32,33,34,35]. This is why we applied the two different heat damage thresholds, or colors: to better interpret the calculated heat values listed in Figure 3c.

**Figure 7:**
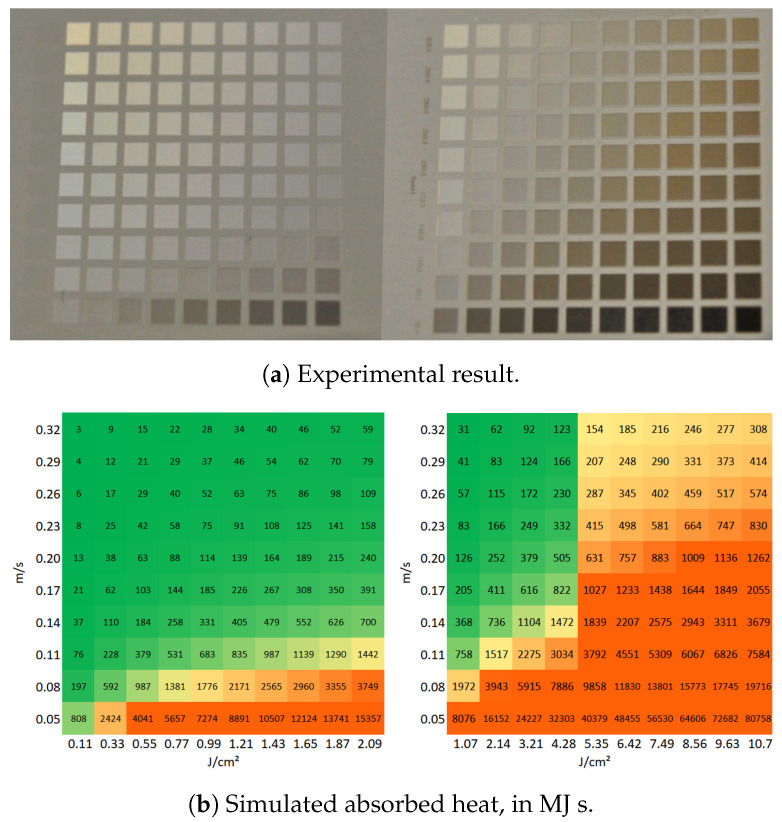
(**a**) Matrices of ablated areas produced on stainless steel 316L using peak fluences in the range between 0.11 J/cm^2^ and 2.09 J/cm^2^ (matrix on the left) and 1.07 J/cm^2^ to 10.7 J/cm^2^, and scanning speeds varying from 50 mm/s to 320 mm/s (bottom to top) at 100 kHz pulse repetition rate, 27 μm spot diameter at focus, 15 μm pitch and 5 layers. (**b**) The simulated absorbed heat color chosen for the low range fluence (F<5 J/cm^2^) is yellow if $C_{td}$ > 1500 MJ s and orange if $C_{td}$ > 4000 MJ s, and green otherwise. For the high fluence range, the areas are yellow if $C_{td}$ > 150 MJ s and orange if $C_{td}$ > 1000 MJ s.

Figure 7 depicts two matrices produced on stainless steel at 100 kHz pulse repetition rate, using fluences in the range between 0.11 J/cm${}^{2}$ to 10.7 J/cm${}^{2}$, scanning speeds in the range between 50 mm/s to 320 mm/s, spot diameter at focus of 27 μm, 15 μm pitch and 5 layers. For this repetition rate, the fluence at which the transition from the low to the high-fluence ablation regime is observed is at about 5 J/cm^2^. At the low-fluence ablation regime, thermal damage is visible for $C_{td}>4000\;MJ\;s$, whereas this value is 1000 MJ s for the high-fluence regime. We also believe that the grayish aspect of the thermally affected areas produced at low fluence, as opposed to the yellowish aspect of the ones obtained at fluences (photos of Figure 7a) may be caused by the different phenomena taking place at each regime during energy absorption and ablation. Moreover, the fact that the values of $C_{{td}_{th}}$ are different for 100 kHz and 500 kHz is also expected since the periods are different and so is the time that the material has to cool down before the subsequent pulse. In any case, once the correlation or calibration between the observed experimental results and the value of $C_{{td}_{th}}$ is done, it can be used as a criterion to predict the appearance of thermal damage in ablated surfaces.

**Figure 8:**
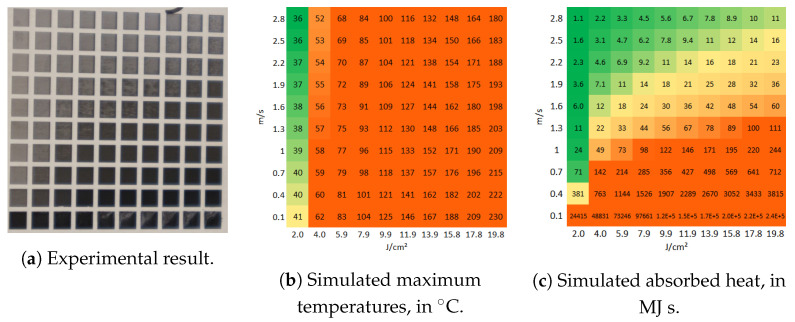
(**a**) Matrix of ablated areas produced on aluminum 2024 using peak fluences in the range between 2 J/cm^2^ and 19.8 J/cm^2^ (left to right) and scanning speeds varying from 0.1 m/s to 2.2 m/s (bottom to top) at 500 kHz pulse repetition rate, 14 μm spot diameter at focus, 5 μm pitch and 50 layers. The area of each ablated square is 1.5 mm × 1.5 mm. (**b**) The simulated temperatures color is orange in the areas at which the temperature exceeds 50 °C. (**c**) The simulated absorbed heat color is yellow if $C_{td}$ > 15 MJ s and orange if $C_{td}$ > 100 MJ s, and green otherwise.

Figure 8a presents a matrix of ablated areas produced on aluminum 2024, using peak fluences in the range between 2 J/cm${}^{2}$ and 19.8 J/cm${}^{2}$ and scanning speeds varying from 0.1 m/s to 2.8 m/s at 500 kHz pulse repetition rate, 14 μm spot diameter at focus, 5 μm pitch and 50 layers. Figure 8b shows the corresponding simulated maximum temperatures reached, considering 28% residual heat. In turn, Figure 8c shows the corresponding estimated absorbed heat. For this alloy, the fluence at which the transition from the low-fluence ablation regime to the high-fluence one is about 4 J/cm^2^. We observe that at 500 kHz, for the low-fluence ablation regime, the material is visibly thermally affected if $C_{td}>10,000\;MJ\;s$, whereas for the high fluence regime, this value decreases to 70 MJ s. These values are higher when working at 100 kHz (Table 2).

**Figure 9:**
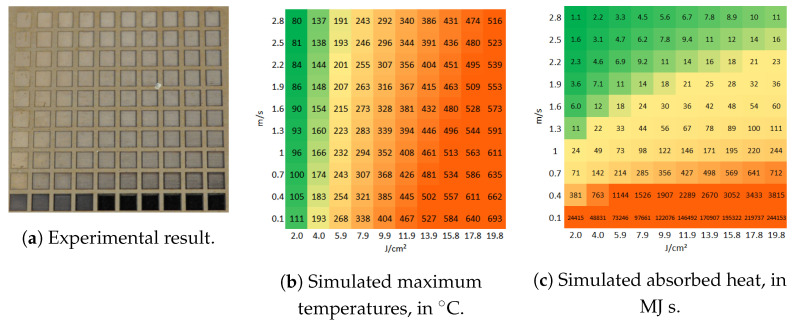
(**a**) Matrix of ablated areas produced on copper using peak fluences in the range between 2 J/cm^2^ and 19.8 J/cm^2^ (left to right) and scanning speeds varying from 0.1 m/s to 2.2 m/s (bottom to top) at 500 kHz pulse repetition rate, 14 μm spot diameter at focus, 5 μm pitch and 50 layers. The area of each ablated square is 1.5 mm × 1.5 mm. (**b**) The simulated temperature color is orange if the temperature exceeds 500 °C are orange. (**c**) The simulated absorbed heat color is yellow if $C_{td}$ > 20 MJ s and orange if $C_{td}$ > 1000 MJ s, and green otherwise.

The processing parameters used in the matrix depicted in Figure 8a were also applied on copper, resulting in the matrix depicted in Figure 9a. Its corresponding simulated maximum temperatures reached are shown in Figure 9b, as well as the estimated absorbed heat (Figure 9c), using a yellow background for the areas at which $C_{td}>20\;MJ\;s$, orange if $C_{td}>1000\;MJ\;s$ and green for lower values. For this metal, the fluence at which the transition from the low-fluence ablation regime to the high fluence one is about 2 J/cm^2^. Similar to stainless steel and aluminum, the absorbed heat threshold at which thermal damage is visible on copper is also higher in the low-fluence ablation regime, but the low and high fluence thermal damage thresholds are the same at 100 kHz and 500 kHz (see listed values in Table 2). The rather high thermal diffusivity of copper may explain why it can manage to conduct heat out of the ablation region similarly and easily at both repetition rates.

The results obtained on copper and aluminum demonstrate that, on the one hand, similar to stainless steel, the maximum attained temperatures simulated at the surface on the ablated workpiece can explain the thermal damage obtained only when analyzing a narrow range of combined fluences and scanning speeds, for instance, when the results obtained at constant speed or constant fluence are compared. Moreover, the results reported in this work show that heat accumulation and thermal damage are linked to the deposited or absorbed summation of energy in time (described by Equation (8)), and there is a direct correlation between the experimental values and the value of $C_{td}$. As a matter of fact, analyzing in this perspective the transient way that heat is absorbed enhances the importance of the irradiation time. The surface of the workpiece may reach elevated temperatures, but if the heating and cooling down cycle is rapid enough, the induced thermal stress may be temporary and return to zero once the temperature again becomes homogeneous. On the other hand, heat-induced phase transformations take place only if the time spent at high temperatures is long enough, as they often depend on the heating and cooling rates [36,37]. Therefore, knowing the maximum temperature reached may not be enough to explain or predict the thermal damage. The threshold value $C_{{td}_{th}}$ depends on the material, its thermomechanical properties and characteristic phase transformation temperatures, and how it absorbs the ultrashort laser energy. These thermal damage thresholds are lower for the aluminum alloy because its melting point is at about 600 °C and its heat capacity is high, even though its thermal diffusivity is higher than that of steel (steel’s melting point is about 1400 °C). Of the three metals studied, copper has the lowest threshold, due to its very high thermal diffusivity plus a high melting point (1084 °C). In any case, the correlation or calibration between $C_{td}$ and the observed thermal damage is straightforward, and the value of $C_{{td}_{th}}$ seems to vary mostly with the fluence window (that determines the ablation regime) and the repetition rate. The accumulated heat calculation is simple, and once the calibration is done, this could potentially be used to predict whether applying a combination of processing parameters may result, or not, in undesirable thermal damage. Such a prediction tool is advantageous if applied in an industrial environment, where rapid process optimization and the reduction of materials consumption required in these type of studies are important.

## 5. Conclusions

In this article, we present a semi-empirical approach to estimate the amount of absorbed heat and correlated it with experimental results obtained on stainless steel 316L, aluminum 2024 and copper. We were able to establish the absorbed heat threshold values above which the material becomes thermally affected, due to the laser treatment, and found that these thresholds vary with the repetition rate and the applied fluence. This approach was compared to an analytical model that allows calculating the transient temperature distribution at the ultrafast laser ablation surface, and the results demonstrate that the here presented semi-empirical model fits better within a wider range of processing parameters.

## Figures and Tables

**Table 1:** Thermal properties of metals used in the calculations.

	κ, m${}^{2}$/s	ρ, kg/m${}^{3}$	$c_{p},$ J/kg K	$T_{\mathrm{melting}},$ °C
AISI 316L	3.9 × 10^−6^	7930	472	1390–1440
Aluminum 2024-T3	49.7 × 10^−6^	2780	875	502–638
Cu	152 × 10^−6^	8818	385	1084

**Table 2:** Transition fluences from low to high fluence ablation regimes, and the absorbed heat thermal damage thresholds determined for 100 kHz and 500 kHz and each fluence range (*L* for low-fluence and *H* for high fluence range) for each of the metals studied.

	Transition Fluence, J/cm${}^{2}$	$C_{{td}_{th},L}$ (100 kHz), MJ s	$C_{{td}_{th},H}$ (100 kHz), MJ s	$C_{{td}_{th},L}$ (500 kHz), MJ s	$C_{{td}_{th},H}$ (500 kHz), MJ s
AISI 316L	5 @ 100 kHz, 1.5 @ 500 kHz	4000	1000	2000	200
Aluminum 2024-T3	4	10,000	3000	10,000	70
Cu	2	10,000	1000	10,000	1000
